# Critical functions of extracellular matrix in brain metastasis seeding

**DOI:** 10.1007/s00018-023-04944-z

**Published:** 2023-09-20

**Authors:** Arseniy E. Yuzhalin, Dihua Yu

**Affiliations:** Department of Molecular and Cellular Oncology, The University of Texas MD Anderson Cancer Center, 6565 MD Anderson Blvd, Unit 108, Houston, TX 77030 USA

**Keywords:** Brain metastasis, Extracellular matrix, Blood, Brain barrier, Integrin, Reelin

## Abstract

Human brain is characterized by extremely sparse extracellular matrix (ECM). Despite its low abundance, the significance of brain ECM in both physiological and pathological conditions should not be underestimated. Brain metastasis is a serious complication of cancer, and recent findings highlighted the contribution of ECM in brain metastasis development. In this review, we provide a comprehensive outlook on how ECM proteins promote brain metastasis seeding. In particular, we discuss (1) disruption of the blood–brain barrier in brain metastasis; (2) role of ECM in modulating brain metastasis dormancy; (3) regulation of brain metastasis seeding by ECM-activated integrin signaling; (4) functions of brain-specific ECM protein reelin in brain metastasis. Lastly, we consider the possibility of targeting ECM for brain metastasis management.

## Introduction

Brain metastasis (BrM) is a devastating complication of several cancer types and a major cause of morbidity and mortality worldwide. Despite advances in cancer treatment, the incidence of BrM continues to rise, affecting about 10% of cancer patients during the course of their disease [1]. BrM is particularly common in patients with melanoma and lung and breast cancers. BrM is manifested by headaches, seizures, cognitive impairment, fatigue, and focal deficits; overall, these symptoms dramatically reduce patients’ quality of life [2]. Unfortunately, the prognosis for patients with symptomatic BrMs remains poor, with a median survival of only 13 months [3].

Current treatment options for patients with BrMs include surgery, chemotherapy, and radiation therapy. All these treatments are mostly palliative and extend patients’ survival by merely a few months. The progress in targeted therapy for BrM is hampered by the lack of BrM-specific, actionable targets. More innovative treatment approaches, such as immunotherapy, are being actively investigated, but the current effectiveness of immune checkpoint blockade monotherapy is very limited in patients with symptomatic BrMs [4–6].

The extracellular matrix (ECM) is a complex network of proteins, glycoproteins, and other molecules that surround and support cells in various tissues throughout the body [7]. The ECM plays a critical role in maintaining tissue structure and function, regulating cell behavior, and modulating cellular responses to environmental cues (Table 1). Further, the ECM acts as a signaling hub, transmitting information from the outside to the inside of cells, and modulating various cellular processes, including proliferation, differentiation, migration, and apoptosis [7]. In addition to its functions in these physiological processes, the ECM plays a key role in the maintenance of tissue homeostasis and repair as well as in pathological processes such as inflammation, fibrosis, and cancer [8]. Fibroblasts are responsible for depositing significant amounts of ECM; as a result, organs with high numbers of fibroblasts, such as the skin, breast, and liver, tend to display an elevated stromal component. It has been proposed that the ECM in these organs may be involved in establishing a pre-metastatic niche [9–11].

**Table 1:** A brief summary of ECM molecules (as proposed by Naba et al. [12])

Domain	Category	Function	Notable examples
Core matrisome	Collagens	Structural	Collagen I, Collagen IV
	Glycoproteins	Structural, functional, signaling	Laminins, Emilins, Tenascins
	Proteoglycans	Functional, signaling	Versican, Perlecan, Biglycan
Matrisome-associated	ECM regulators	Regulatory (ECM-remodeling enzymes, crosslinkers, proteases, regulators etc.)	Lysyl oxidases, Serpins, Cathepsins
	Secreted factors	Known or suspected to bind core ECM proteins	CXCLs, Angiopoietins, S100A

**Fig. 1:**
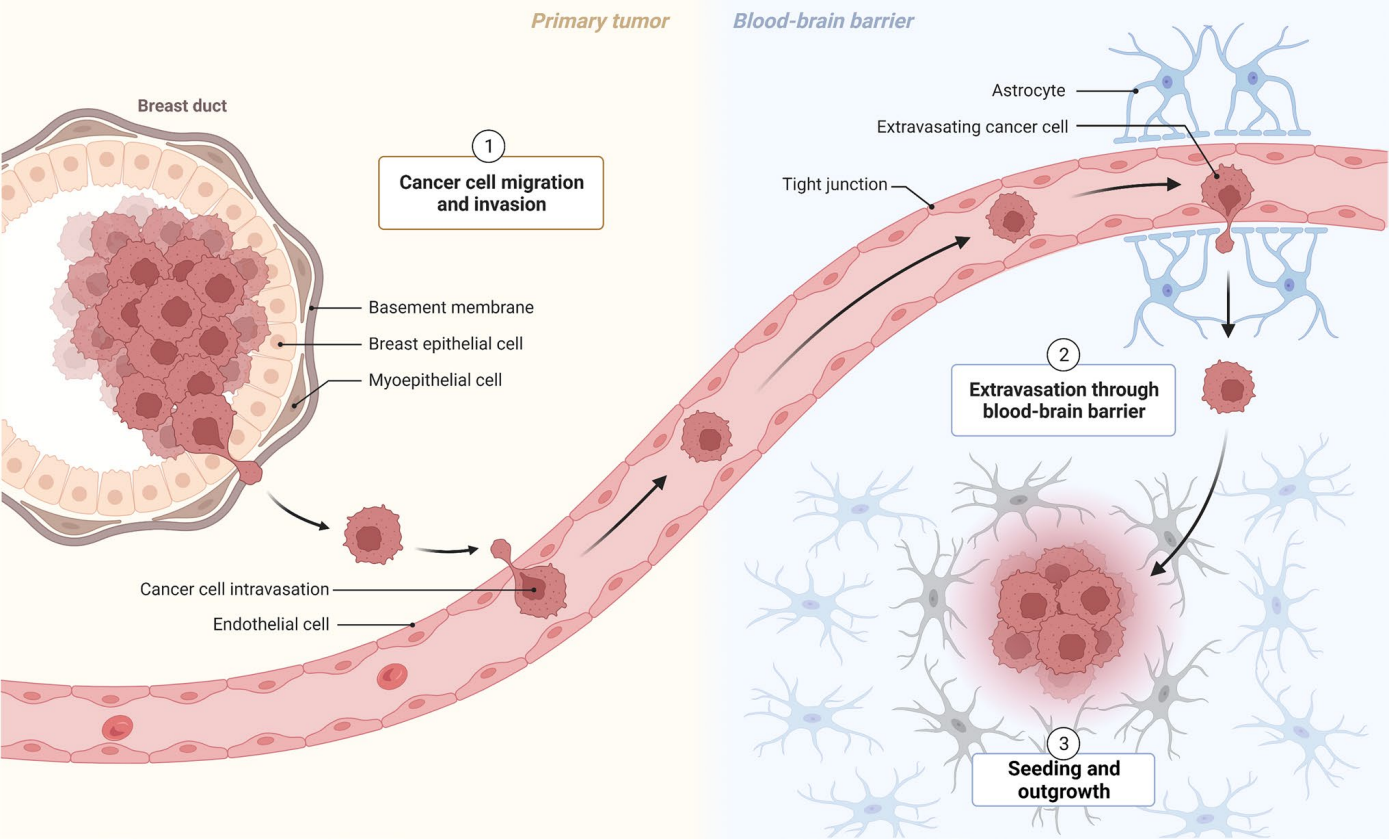
Schematic explaining the process of BrM establishment. Adapted from “Breast Cancer To Brain Metastasis”, by BioRender.com (2023). Retrieved from https://app.biorender.com/biorender-templates

To successfully develop BrMs, cancer cells must adapt and survive in the hostile, nutrient-deficient microenvironment of the brain (Fig. 1). Examples of BrM-specific adaptations include activation of oncogenic kinases [13], induction of PTEN-loss [14], establishing an immunosuppressive microenvironment [15], increasing oxidative phosphorylation [16], competition for limited resources such as iron [17], synthesis of fatty acids [18], and induction of vascular co-option [19]. In recent years, substantial evidence indicates the involvement of ECM of distant organs in metastasis [20–22]. The ECM has been implicated in regulating attachment of metastasizing cancer cells [11, 23], controlling metastasis dormancy [24], and mediating the immune escape of metastasis [25]. However, knowledge about the role of the ECM in BrMs is scarce and has not been systemically assessed. In this article, we provide a comprehensive overview of studies investigating the involvement of the ECM in the incidence and pathogenesis of BrM.

## Brief overview of brain ECM

Securely contained in the cranium, the brain is one of the softest organs in the human body because of minimal concentrations of fibrous ECM, the main contributor of tensile strength in an organ [26]. Approximately 60% of adult human brain is fat, whereas the remaining 40% is water, protein, carbohydrates, and salts. Only ~ 20% of adult brain accounts for the extracellular space, and ECM proteins constitute only a fraction of that amount [27]. Whereas stiffer organs contain an abundance of collagen and elastin, the brain ECM backbone is primarily composed of a disaccharide polymer, hyaluronan (HA), which is a much softer matter [28]. The evolution of the brain has dramatically altered the function and structure of its ECM, which can be categorized into three major compartments: the blood–brain barrier (BBB), perineuronal net (PNN), and neural interstitial matrix [29]. In addition to these categories, some researchers also define perinodal ECM, a separate class of brain matrix that surrounds nodes of Ranvier within axons and whose protein composition resembles that of the PNN [30].

The BBB is formed by endothelial cells that compose the walls of the capillaries, which are ensheathed by basement membrane primarily consisting of type IV and XVIII collagens, laminins, nidogens, and heparin sulfate proteoglycans [31]. Capillary basement membrane is a prime physical barrier protecting the neural tissue from infiltrating cells and chemical compounds, and its breakdown is fraught with BBB dysfunction, which ultimately may lead to instability of the central nervous system and the onset of neurological diseases [32]. However, it is important to note that capillary basement membrane is not the sole type of barrier comprising the BBB, which also includes tight junctions between adjacent endothelial cells; a transport barrier, that is, specific transport mechanisms mediating the flux of ions; and a metabolic barrier, consisting of enzymes that metabolize molecules in transit [33].

The PNN is a scaffold primarily consisting of HA, connecting surrounding neurons and proximal dendrites into a net-like fibrous structure. Besides HA, the PNN consists of tenascins C and R as well as link proteins and chondroitin sulfate proteoglycans, all of which stabilize HA. The exact function of the PNN is incompletely studied; however, it has been strongly implicated in maintaining synaptic plasticity [34, 35] and the formation of long-term memories [36]. In addition, PNNs were reported to protect brain cells from oxidative stress [37] and neurotoxins such as amyloid beta-protein [38]. The perinodal ECM has a very similar structure to the PNN: its core component is HA, which is stabilized by hyaluronan and proteoglycan link protein 2 (HAPLN2) as well as brevican, versican, and neurocan [30]. The primary function of the perinodal ECM is to facilitate the formation of nodes of Ranvier and modulating axon conduction by regulating extracellular electrical resistance [30].

The neural interstitial matrix is an ensemble of all the remaining ECM constituents loosely dispersed in the brain parenchyma. Major components of the neural interstitial matrix are proteoglycans, HA, tenascins, link proteins, collagen XV, elastin, laminin 511, and fibronectin [39]. The function of the neural interstitial matrix is understudied; although it does not provide major structural support to the brain due to its scarcity, it may represent residual structures formerly in control of brain development. Matrix molecules, especially fibronectin, play a dominant role in development of the brain and constitute up to 40% of its mass in the perinatal period [29, 40]. In the postnatal period, the ECM proportion in the brain reduces by twofold. It is possible that individual constituents of the neural interstitial matrix exert special signaling functions, facilitating neuronal function. For example, the ECM glycoprotein reelin directly controls the migration of neurons in the central nervous system and regulates dendritic morphogenesis and neurotransmission [41].

**Fig. 2:**
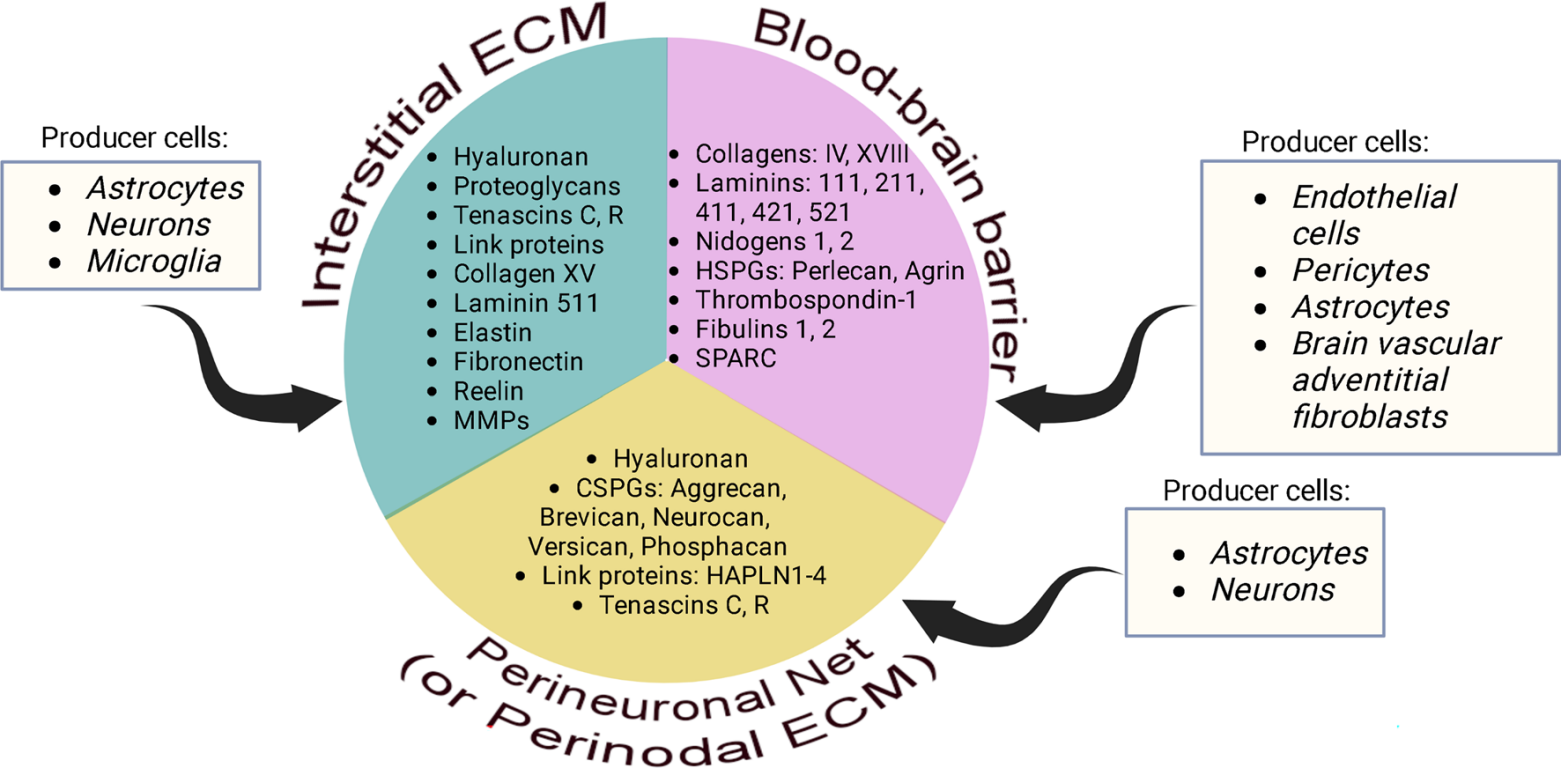
Overview of major ECM constituents of an adult human brain as well as cell types producing them. Abbreviations: *HSPGs* heparan sulphate proteoglycans, *CSPGs* chondroitin sulfate proteoglycans, *HAPLN* hyaluronan and proteoglycan link protein, *MMPS* matrix metalloproteinases, *SPARC* secreted protein acidic and rich in cysteine, *ECM* extracellular matrix. Created with BioRender.com

Brain matrisome proteins come from many different sources. One of the largest producers of ECM in the brain are astrocytes (glial cells), which deposit HA, proteoglycans, and tenascins into the extracellular space [42, 43]. In addition, astrocytes produce versican V2 and brevican for the formation of nodes of Ranvier [30]. Microglia, in contrast, clear the redundant ECM by either secreting proteolytic enzymes or directly engulfing and phagocytosing unwanted ECM components [43]. Neurons have been reported to produce tenascin-C, tenascin-R, and the chondroitin sulfate proteoglycans brevican and neurocan, all of which were shown to be required for establishment of the PNN and synapse formation [44]. Neurons also synthesize reelin, a major ECM chemoattractant and signaling molecule [45]. Various matrix proteins comprising the inner and outer vascular basement membranes of the BBB are produced by cerebral endothelial cells and pericytes and, to a lesser extent, by astrocytes and brain adventitial fibroblasts [33, 46]. It should also be noted that when needed, virtually all brain cell populations have an ability to remodel the surrounding ECM by producing matrix-specific proteolytic enzymes (e.g., MMPs, cathepsins, ADAMs) and their inhibitors (e.g., TIMPs) [43]. Thus, all the brain cell types are involved in production and maintenance of the ECM (Fig. 2).

## Disruption of the BBB in BrMs

Early BrMs are located in the brain perivascular niche, where they arrest exclusively in brain capillaries and/or postcapillary venules [47]. Successful dissemination to the brain parenchyma would require cancer cells to partially disintegrate the BBB. Early studies on this topic, led by Josh Fidler, showed that the BBB’s permeability was compromised in several experimental BrM models *in vivo* [48]. Later, it was demonstrated that brain-seeking 4T1 mammary tumor cells disintegrate BBB through disruption of ZO-1 and claudin-5 tight junctions in vivo [46]. In this study, the frequency of BBB-penetrating cancer cells coincided with increased permeability of the BBB, which indicates that BrM seeding is an active, not passive, process directly linked to tight junction breakdown [49]. Several studies indicated that BBB disruption is a *modus operandi* for metastasizing cells from triple-negative breast cancer (TNBC) and basal-type breast cancers but not those from HER2/neu-positive breast cancer [50, 51], but the reason for this difference is currently unknown. Our group has found that breast cancer cells harboring activated Src tyrosine kinase can induce severe BBB disruption [13]. Metastatic cells have also been reported to disrupt the BBB through secreting a protease cathepsin [51]. Genetic knockout of the *CTSS* gene encoding cathepsin S in brain-seeking MDA-MB-231 cells led to a dramatic reduction in metastatic seeding in the mouse brain upon their intracardiac injection [51]. It was further shown that tumor cell-derived cathepsin S actively cleaves tight junction proteins that regulate BBB integrity [51]. In addition to directly secreting ECM-remodeling factors, cancer cells are able to produce exosomes loaded with lnc-MMP2-2 RNA, which promotes BBB disruption in vivo by modulating human brain microvascular endothelial cells [52]. The same study showed that lnc-MMP2-2 functions as a molecular sponge for miR-1207-5p, which is critical for production of tight junction proteins in human brain microvascular endothelial cells, by upregulating *EPB41L5*. Thus, metastasizing cancer cells can “educate” major BBB-producing cells (i.e., endotheliocytes, astrocytes, etc.) to break down tight junctions and enable further dissemination of BrMs.

These findings from in vivo studies are further supported by human pathology research. Remodeling of the ECM by BrMs was evident from study examining 40 patients with lung and breast cancer-derived BrMs [53]. At both mRNA and protein levels, more than 80% of lung cancer-derived BrMs and 50% of breast cancer-derived BrMs expressed MMP14 [54], a known indicator of poor prognosis in cancer [55–57]. Of note, the authors observed a negligible expression of MMP7 in the BrM lesions [53]. Whether MMP14 plays a direct role in BBB breach remains an open question requiring further investigation.

Multiple studies have demonstrated the contribution of BBB-associated heparan sulfate proteoglycans (HSPGs) in the onset of BrMs. HSPGs are glycoproteins containing covalently attached heparan sulfate chains, a type of glycosaminoglycan (GAG) [58]. Their major functions include: (i) cooperating with integrins to facilitate cell–cell and cell–matrix communications, (ii) storing growth factors and soluble ECM-affiliated molecules which will be released upon HSPG degradation, (iii) defining the basement membrane structure (as a part of BBB), and (iv) acting as receptors and co-receptors for different tyrosine kinase-type growth factor receptors. In the BrM setting, tumor astrocytes were shown to produce large amounts of heparanase, an endoglycosidase which cleaves HSPGs [59]. Furthermore, treatment of BrM cancer cells with astrocyte conditioned media containing heparanase dramatically promoted their migration [60]. Intriguingly, highly brain-tropic human melanoma cells degraded purified ECM HSPG and HSPG cell-surface subpopulations faster than sublines of lower metastatic potential [61], indicating that HSPGs indeed pose a major barrier to metastasizing tumor cells. Accordingly, inhibition of cancer cell invasion by heparanase blockade was further confirmed in pediatric brain tumor cells *in vitro* [62].

Metabolism of HA can help establishing the brain metastatic niche through controlling the integrity of the BBB. Hyaluronidase 1 (HYAL1), an enzyme that cleaves HA into low molecular weight fragments, was shown to be produced by BrM-initiating cells, and it could mediate the adhesion of BrM cells to primary human brain endothelial cells *in vitro* [63]. Furthermore, it was found that the *HYAL1* gene knockout in BrM cells led to a reduced BrM burden after intracardiac injection, compared to vector control cells [63]. Thus, pericellular HA-coat may facilitate the tumor cell interaction with the brain endothelium and lead to successful BrM seeding of disseminated cancer cells.

Finally, it was reported that the BBB can be selectively disrupted in BrMs [64] at least in part due to inhibition of major facilitator superfamily domain 2a (Mfsd2a), the endothelial cell-expressed docosahexaenoic acid transporter that is critical for the formation and function of the BBB [65]. Compared with normal brain, the expression of the *Mfsd2a* gene was markedly decreased in BrMs derived from breast cancer, lung cancer, or neuroendocrine prostate cancer. Importantly, BrM-associated vasculature demonstrated a similar dramatic reduction in the expression of the *Mfsd2a* gene compared to the normal brain vasculature [64]. The same study further demonstrated that perivascular astrocytes promote *Mfsd2a* expression in endothelial cells through secreting TGFβ and bFGF, while metastasizing tumor cells can disrupt this signaling pathway to downregulate *Mfsd2a* and thus facilitate BBB degradation [64].

Taken together, these studies indicate that metastasizing tumor cells employ a plethora of means to break down the BBB to extravasate and seed in the brain. Notably, brain-tropic cancer cells can degrade the BBB on their own or through “educating” cells in the brain microenvironment, such as astrocytes.

## ECM molecules in control of BrM dormancy

The vast majority of cancer patients (> 90%) have resting-state (G0) tumor cells in the brain [66]; these dormant cancer cells may take years or even decades to awake and outgrow as clinically detectable BrMs [67]. Both entering and exiting a dormant state require a permissive milieu, and identification of mechanisms responsible for these events poses a great challenge for scientists. The biology and mechanisms of cellular dormancy in BrM remain incompletely understood, yet it has been shown that patients with BrMs derived from dormant disseminated cells have a longer median survival [68].

Recent studies have demonstrated a key role of the astrocyte-derived ECM in regulating the dormant state of disseminated tumor cells in the brain [69]. During the early steps of BrM progression, astrocytes are stripped from the vasculature and segregated to the border of BrMs. Such eviction of astrocytes coincides with removal of their end-feet as well. Upon contact with disseminated tumor cells, astrocytes then induce their dormancy via deposition of laminin-211 into the parenchymal basement membrane, which transduces pro-dormancy signaling via receptor dystroglycan [69]. It is interesting that integrins, classically recognized laminin receptors, are not involved in this pro-dormancy signaling [69]. Further mechanistic studies revealed that laminin-211–activated dystroglycan promotes BrM cell dormancy by tethering Yes-associated protein (YAP) to the cell membrane [69]. YAP is a major Hippo pathway effector [70] previously linked with lung cancer cell dormancy through repressing the pro-apoptotic protein Bmf via Slug. In the BrM setting, cancer cells with constitutively active YAP, which shuttled freely to the nucleus and back, can escape from dormancy and accelerate brain colonization *in vivo* [69]. Activation of dystroglycan by astrocyte-derived laminin-211 on the surface of cancer cells resulted in sequestering YAP to the cancer cells’ membrane and promoting the quiescent phenotype in experimental models of mouse BrM [69]. These findings suggest that BrM-associated astrocytes may limit the growth of BrM during early steps of metastatic dissemination, and that escape from BrM dormancy can be triggered by factors that would enable the drift/relocation of brain astrocytes from dormant cells, such as neuroinflammation or trauma. However, it remains unclear whether these findings are clinically relevant, as astrocytes have been widely reported to secrete multiple pro-tumor factors that promote BrM growth, stemness, invasiveness, and chemoresistance [71].

**Fig. 3:**
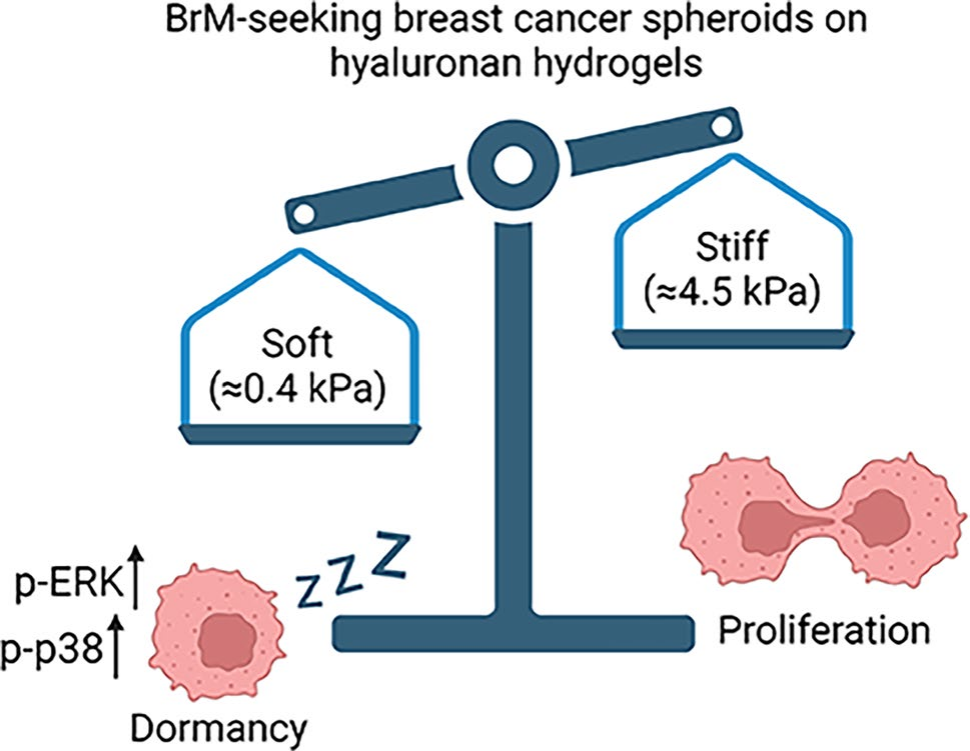
ECM softness controls dormancy state in BrM-seeking cells. Created with BioRender.com

The crosstalk between dormant cancer cells and surrounding ECM can be investigated using hydrogels, representing an engineered native-like ECM structure [72]. In one such study, brain-tropic MDA-MB-231 and BT474 cells, grown as 3D spheroids in HA-based hydrogels, demonstrated a reduction in proliferation marker Ki67 and a higher percentage of cells positive for dormancy marker phospho-p38, compared to spheroids grown in an ECM-free environment [73]. Importantly, transferring dormant spheroids into a scaffold-free culture system reinstated spheroid growth and aborted the dormancy program [73]. These results indicate that the ECM can, in principle, induce dormancy of brain-tropic cells. A similar study investigated the composition of ECM-like hydrogels on the behavior of brain-tropic MDA-MB-231 subline BrM2a-831 [74]. The authors formulated hydrogels of varying degradability and adhesiveness and identified the ECM parameters with the highest impact on cancer cell dormancy in vitro. In particular, they demonstrated that while hydrogels with high adhesivity and degradability promoted cancer cell proliferation, those with no adhesivity but high degradability had a potential to induce dormancy and activate survival programs in BrM2a-831 cells as well as in bone- and lung-tropic MDA-MB-231 sublines [74]. Importantly, the authors could reactivate dormant cells by increasing hydrogel adhesivity in vitro, which enabled the cells to exit dormancy [74]. These results are consistent with a study showing that soft HA-based hydrogels can induce a much stronger dormancy phenotype (as measured by Ki67^+^ cells) in brain-tropic MDA-MB-231 cells than can stiff hydrogels [75]. Thus, ECM degradability/softness plays a key role in inducing dormancy, whereas its adhesiveness/stiffness may help dormant cells to re-enter the cell cycle (Fig. 3). Further studies to validate these exciting findings in vivo and in human disease are warranted.

## ECM-activated Integrin signaling facilitates BrM seeding

**Fig. 4:**
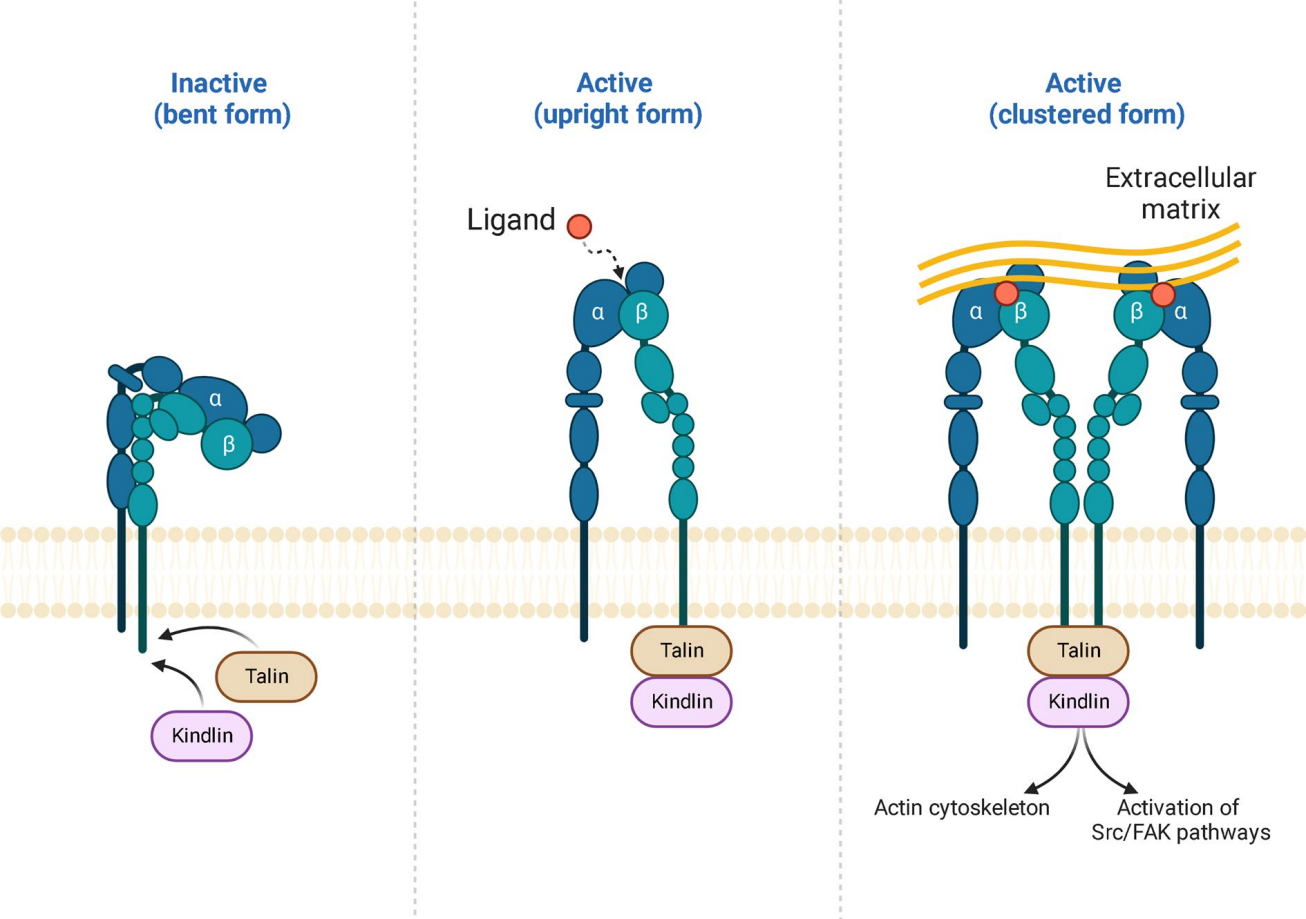
Schematic illustration of integrin structure and activation. Signals both inside and outside the cell can trigger integrin activation, which induces multiple signaling pathways to regulate cytoskeleton assembly, adhesion, migration, proliferation, etc. Reprinted from “Integrin Structure and Activation”, by BioRender.com (2023). Retrieved from https://app.biorender.com/biorender-templates

Integrins are a class of molecules primarily responsible for “outside-in” signaling in control of communication between the ECM and surrounding cells [76]. Integrins are composed of two subunits, α and β, which form a heterodimeric structure [77]. There are 18 α and 8 β subunits, which can form 24 distinct integrin receptors, each with unique binding properties and functions [78]. Integrins have multiple roles in the cell; however, they are not constitutively active and require “outside-in” signals from ECM proteins to activate them. Integrins exist in low-affinity “bent” conformation when inactive. Upon contact with an ECM ligand, integrin’s ectodomain turns into “upright” conformation, leading to activation of a cytoplasmic tail of the β subunit, which binds several intracellular anchor proteins, including talin, kindling, etc [78, 79]. If stronger activation is needed, extended integrins can further cluster at cell-ECM contacts to create the foundation for adhesion complexes [80]. Thus, integrins act as signaling receptors, transmitting signals bidirectionally across the cell membrane to control multiple cellular processes (Fig. 4). Dysregulation of integrin function has been implicated in cancer [81], autoimmune disorders [82], and cardiovascular diseases [83].

Certain ECM proteins in the brain perivascular niche could facilitate the adaptation of disseminating cancer cells in the brain. Profiling of the BrM-initiating capability of different cancer cell lines in vivo revealed that human lung cancer line EBC-1 had the highest probability to induce BrM (~ 100%) when injected via the intracardiac route [84]. Follow-up analysis of integrin chains reveled that a BrM-derived EBC-1 subline expressed significantly higher levels of integrin α3 than did parental or bone–homing EBC-1 counterparts [84]. In vitro, this BrM-derived EBC-1 subline exhibited a more pronounced attachment to type I collagen, fibronectin, and laminin, all of which are binding partners of α3β1. Interestingly, the BrM capacity of brain-seeking EBC-1 subclones was impaired upon in vivo treatment with an integrin α3–blocking antibody [84]. While this study implicated integrin α3 in control of BrM formation, another study showed that 4T1 mouse mammary tumor cells with knockdown or pharmacological inhibition of integrin α3 displayed a dramatic reduction of lung metastasis in a tail vein injection model [85], indicating that integrin α3 is also critical for metastasis to other organs. Since cancer cells injected into the tail vein rarely metastasize to the brain, this study is limited in assessing the capability of 4T1 cells to metastasize to the lungs only. Nonetheless, in the BrM setting, the function of integrin α3β1 has been explained by its interaction with αB-crystallin, a molecular chaperone responsible for skeletal myopathies [86] and cardiomyopathies [87]. It was shown that αB-crystallin increases adhesion of TNBC cells to human brain microvascular endothelial cells through an integrin α3β1-dependent mechanism and also promotes adhesion to type I collagen, fibronectin, and laminin [88].

In addition to their role in BrM seeding, integrins can also serve as biomarkers of, or therapeutic targets for established BrMs. Berghoff et al. [89] classified invasion patterns in established human BrMs and identified three subtypes characterized by well-demarcated borders, vascular co-option, or diffuse infiltration. Profiling of integrin expression in these subtypes revealed that integrin αvβ6 was significantly overexpressed in the well-demarcated group compared with the vascular co-option and diffuse infiltration groups [89]. Theoretically, well-demarcated BrMs without loci of aggressive infiltration may take a significantly longer time to grow and become symptomatic. In support of this hypothesis, the expression of integrin αvβ6 correlated with lower cell proliferation index in BrMs [90]. Lastly, the expression of integrin αvβ6 in BrMs was associated with a longer overall patient survival after BrM diagnosis [90]. Thus, integrin αvβ6 is a feature of well-demarcated BrMs characterized by slower growth, which could be used in prognostication for BrM patients. Importantly, targeting of integrin αvβ6 by an antipsychotic drug, penfluridol, showed promising results in treating TNBC BrMs *in vivo* [91], suggesting a strong clinical relevance of this BrM-associated integrin. Inhibition of other αv integrin chains has also been examined in preclinical trials. Wu et al. [92] treated rat BrMs from brain-tropic MDA-MB-231. HER2 subline with intetumumab, an antibody binding αv integrins. The authors observed that intetumumab prevented metastasis formation in 30% of tested rats while the remaining animals displayed a reduction in BrM burden compared to controls [92]. Thus, inhibition of integrins, particularly αv chains, may represent an attractive strategy to treat BrMs.

## Pivotal role of Reelin in BrM and central nervous system metastasis

Reelin is a major brain-specific ECM glycoprotein involved in cortical development and synaptic function maintenance [93]. Deposited in the extracellular space, reelin binds to its receptor apolipoprotein E receptor 2 (ApoER2) expressed on the cell membrane of neurons. This leads to activation of a downstream signaling cascade that eventually promotes Ca2^+^ influx and subsequent release of neurotransmitters. Notably, mutations in the reelin-encoding gene, *RELN,* are linked to schizophrenia in women [94], autism [95], and Alzheimer disease [96], highlighting the importance of this glycoprotein in normal brain physiology. Even though reelin’s expression is restricted to brain cells, recent studies showed that BrM cancer cells can acquire a neuronal-like metaprogram and express brain-specific proteins to adapt to the microenvironment of the brain [97]. Therefore, it is possible that neuron-like BrM cancer cells can also express reelin. Indeed, it was found that brain-tropic breast cancer cells produce more reelin compared to their parental counterparts [98]. Furthermore, dormant brain-tropic breast cancer sublines of MDA-MB-231 and BT474 overexpress reelin after co-culture with neurons [88, 99]. The knockdown of the *RELN* gene resulted in downregulation of key synaptic mediators *NTRK2*, *NGFR*, *NRXN1*, and *NLGN4X*, suggesting that neuronal exposure enhances reelin expression in BrM cells to activate a neuron-like phenotype [98].

Human epidermal growth factor receptor 2 (HER2) overexpression (HER2^+^) is frequently (15–20%) detected in breast cancer [100], and patients with HER2^+^ breast cancer have an increased risk of developing BrMs [101]. A recent study showed that reelin is highly expressed in HER2^+^ BrMs compared to TNBC BrMs, and that reelin co-localizes with HER2 in BrM tissue Sects.  [99]. Furthermore, co-immunoprecipitation studies revealed that reelin can form a complex with HER2 [102], implicating a functional link between these two proteins. It was further shown that reelin knockdown inhibited the spheroid-forming ability of BrM-capable HER2^+^ cells but not TNBC cells [99]. Interestingly, the authors found the reelin-expressing BrM cells are associated with astrocytes and they also showed that conditioned media from astrocytes can induce reelin in HER2^+^ BrM cells [99], suggesting that not only neurons but also glial cells are able to promote reelin expression in BrM. The functional role of rellin-HER2 complex in BrMs warrants further investigations.

Reelin has recently been implicated in promoting metastasis in medulloblastoma, a rare pediatric tumor of the cerebellum, that metastasizes almost exclusively to the spine or intracranial leptomeninges [103]. The authors of that study identified that overexpression of SMARCD3 in medulloblastoma activates reelin–DAB1–Src signaling-mediated cancer cell migration, which eventually results in metastatic dissemination [104]. In this pathway, the role of reelin is pivotal, as reelin was found to be directly regulated by a key epigenetic modulator, SMARCD3, to launch a cascade of factors that trigger cancer cell metastasis [104]. Thus, it appears that reelin may be a key ECM protein involved in central nervous system metastasis.

## Discussion and concluding remarks

BrM remains an unmet clinical need, with devastating incidence rates of 2.8–14.3 per 100,000 people of general population [105]. The advent of single-cell sequencing and spatial profiling in recent years enabled a deeper understanding of BrM mechanisms and pathophysiology, even though this has not yet led to qualitative improvements in treatment. The importance of ECM in progression of primary tumors and metastasis has long been underestimated for several reasons, including the fact that ECM is difficult to extract and investigate owing to crosslinking and insolubility and also because ECM research is at the intersection of cell/molecular biology and biophysics that requires multidisciplinary approaches. In this review, we summarized knowledge about BrM-associated ECM to highlight its significance in the development of disease.

**Fig. 5:**
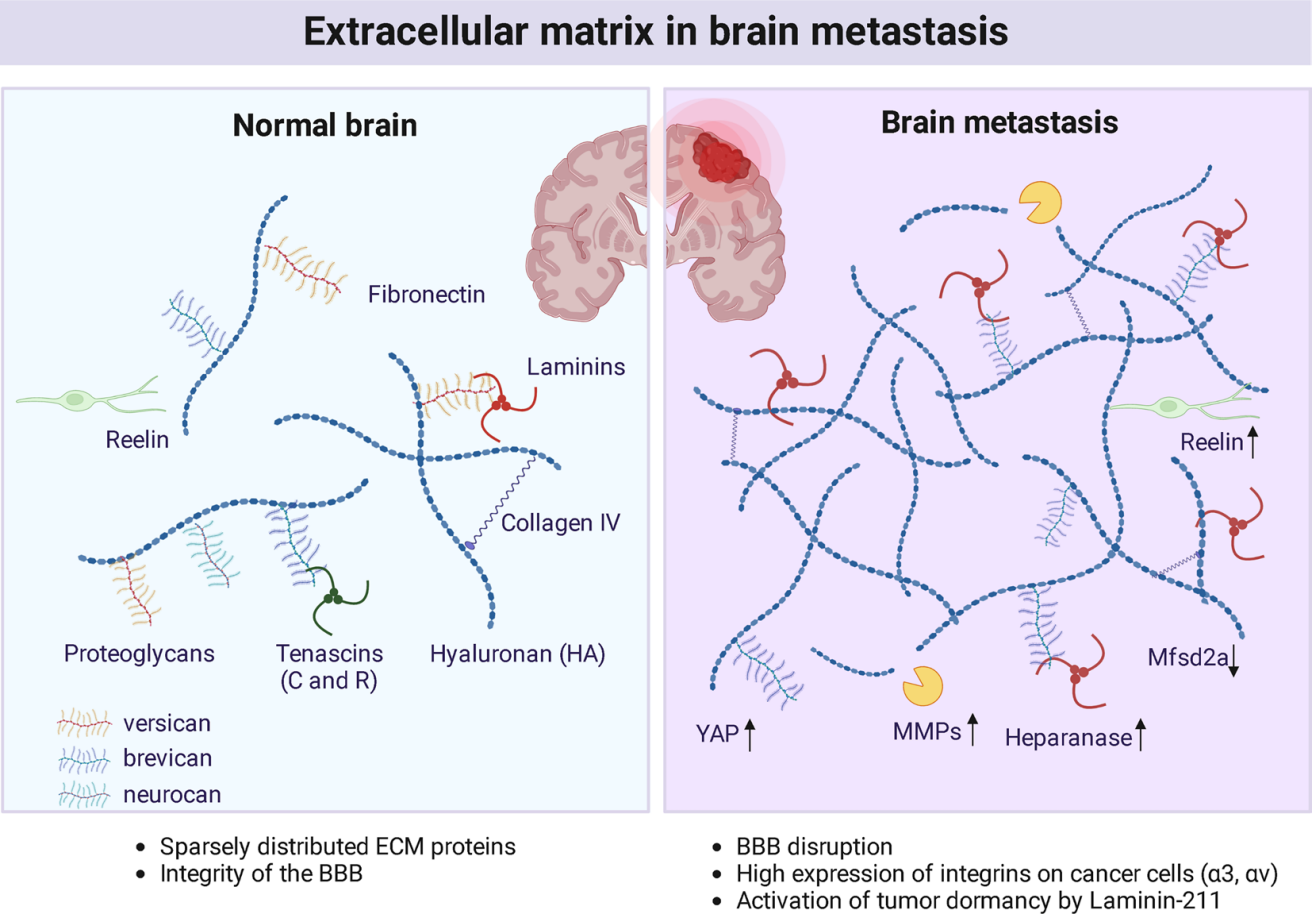
Features of ECM in a healthy normal brain versus brain metastasis. Normal brain is characterized by sparsely distributed ECM proteins as well as high integrity of the BBB. In brain metastasis, the BBB is disrupted through Mfsd2a downregulation in epithelial cells. Deposition of Laminin-211 by astrocytes promotes cancer cell dormancy via YAP signaling. Cancer cells produce large amounts of integrins (α3, αv) on the cell surface. Metastasis-associated ECM has high concentrations of MMPs, Heparanase, and Reelin. Adapted from “Extracellular Matrix in Brain Tumorigenesis”, by BioRender.com (2023). Retrieved from https://app.biorender.com/biorender-templates

The brain is notable for poor ECM content, mainly due to the lack of fibroblasts, except for brain vascular adventitial fibroblasts. The matrisome of the normal brain is limited to only 69 proteins, among which are only nine proteoglycans and 18 collagens [106]. Nonetheless, the brain ECM plays a major role during development [29] and maintains BBB integrity [31], highlighting the ECM’s unique function in this organ. Importantly, cerebrovascular matrisome may be completely different to the ensemble of ECM proteins produced by metastasizing cancer cells; therefore, when studying the BrM-associated ECM, it is particularly important to identify the source of studied ECM proteins, whether they derive from the brain cells or the tumor cells. In our studies, we observed that in experimental mouse models of BrM, some mouse mammary tumor cell lines produce virtually no ECM (such as EO771 or 4T1), whereas the others display extensive ECM deposits (EMT3). In agreement, a comparative analysis of metastatic matrisome from different organs revealed that BrMs had at least fourfold lower abundance of matrisome proteins compared to metastases to other organs [107]. The exact function of BrM-derived ECM proteins remains to be determined in future studies (Fig. 5).

This review has several limitations. Even though this article does not cover the role of immune cells in BrMs, recent studies have demonstrated a complex interaction between tumor-associated ECM and tumor-infiltrating leukocytes, monocytes/macrophages, and granulocytes [108]. In the context of BrMs, collagen deposits were co-localized with CD3^+^ T cells and CD68^+^ macrophages [109], so it would be interesting to explore whether BrM-associated ECM may influence immune cell trafficking, antigen recognition, presentation, or cancer cell killing. In addition, this review predominantly covered biochemical cues derived from brain ECM in cancer metastasis; however, biophysical signals are also an important factor to regulate cancer cell dormancy and colonization. Due to the dearth of published studies, this aspect remains poorly understood and warrants further investigation. 

Certain components of the tumor microenvironment may be successfully targeted to treat solid tumors. Immunotherapies or anti-angiogenic therapies using monoclonal antibodies to inhibit key proteins in the pro-tumor microenvironment (e.g., PD-L1, VEGF) have achieved dramatic improvement in survival of patients with cancer [110, 111]. Studies discussed in this review raise the possibility of targeting specific brain ECM components for BrM treatment (Tables 2 and 3); this is further supported by our finding that upregulation of ECM genes is generally associated with poor tumor outcomes across multiple cancer types [112], suggesting the potential benefit of normalizing the ECM. However, even though numerous clinical trials were conducted during the past decade to inhibit ECM synthesis or enhance its turnover, these attempts did not bear fruit. Blockade of collagen-crosslinking enzyme lysyl oxidase did not show any survival benefit in pancreatic cancer [113] or colorectal adenocarcinoma [114], yet initial preclinical trials were very optimistic [115]. Furthermore, targeting integrins through FAK inhibition did not improve the outcome of patients with mesothelioma [116]. Along similar lines, breakdown of HA by delivering hyaluronidase in patients with pancreatic cancer did not prove to be effective [117]. Attempts to facilitate the remodeling of tumor-associated ECM by MMP inhibitors failed because of limited effectiveness [118]. The lack of success in these trials of targeting the ECM may be explained by the complexity of ECM and our poor understanding of how/when individual ECM components are promoting cancer progression. In the context of BrM, the situation is additionally complicated by the BBB, which restricts the delivery of many therapeutics to the brain. It has been postulated that permeabilizing the BBB in patients with symptomatic BrMs could facilitate the delivery of drugs and enhance therapeutic efficacy [119]. On the other hand, novel therapies targeting the ECM such as gene therapies that strengthen the BBB’s integrity, may be effective in preventing BrMs in high-risk patients. For example, adeno-associated viruses designed to selectively target cells of the brain endothelium would carry transgenes such as *Mfsd2a* and be used to increase BBB integrity to build up protection from circulating tumor cells’ entering the brain. Additionally, combining ECM-targeting therapies with other BrM-inhibiting and brain microenvironment-normalizing therapies may lead to a better control of BrM progression. Clearly, disruptive research on BrM-associated ECM is needed to bring about substantially improved treatment for BrM patients in dire need.

**Table 2:** A brief summary of ECM molecules (as proposed by Naba et al. [12])

Domain	Category	Function	Notable examples
Core matrisome	Collagens	Structural	Collagen I, Collagen IV
	Glycoproteins	Structural, functional, signaling	Laminins, Emilins, Tenascins
	Proteoglycans	Functional, signaling	Versican, Perlecan, Biglycan
Matrisome-associated	ECM regulators	Regulatory (ECM-remodeling enzymes, crosslinkers, proteases, regulators etc.)	Lysyl oxidases, Serpins, Cathepsins
	Secreted factors	Known or suspected to bind core ECM proteins	CXCLs, Angiopoietins, S100A

**Table 3:** Potential ECM targets for BrM therapy and prevention

ECM component	Targeted therapy potential
Heparanase	To prevent BrM seeding [59, 60, 62]
Hyaluronidase 1	To prevent BrM seeding [63]
Cathepsin S	To prevent BrM seeding [51]
Laminin-211	To prevent BrM seeding [69]
Dystroglycan	To prevent BrM seeding [69]
Integrin α3	To prevent BrM seeding [84]
Integrin α3β1	To prevent BrM seeding [88]
Integrin αvβ6	To reduce BrM growth [91]
αv integrins	To prevent BrM seeding [92]
Reelin	To prevent early BrM adaptation by acquiring a neuron-like phenotype [98, 99]
	To limit growth of HER2^+^ BrMs [99]
	To prevent metastasis from cerebellum to other parts of the brain [104]

## Data Availability

Not applicable.
